# The Tendency to Avoid Physical Activity and Sport Scale (TAPAS): Rasch analysis with differential item functioning testing among a Chinese sample

**DOI:** 10.1186/s40359-023-01377-y

**Published:** 2023-11-04

**Authors:** Chia-Wei Fan, Yen-Ling Chang, Po-Ching Huang, Xavier C. C. Fung, Ji-Kang Chen, Nadia Bevan, Kerry S. O’Brien, Ya-Chin Yeh, Hsin-Pao Chen, I-Hua Chen, I-Ching Lin, Mark D. Griffiths, Chung-Ying Lin

**Affiliations:** 1 Department of Occupational Therapy, AdventHealth University, 671 Winyah Drive, Orlando, FL 32803 USA; 2 Department of Family Medicine, Cardinal Tien Hospital, No. 362, Zhongzheng Rd., Xindian Dist, New Taipei, 231403 Taiwan; 3 Institute of Allied Health Sciences, College of Medicine, National Cheng Kung University, No. 1, University Rd., East Dist, Tainan, 701401 Taiwan; 4 Department of Rehabilitation Sciences, Faculty of Health and Social Sciences, The Hong Kong Polytechnic University, 11 Yuk Choi Rd., Hung Hom, Kowloon, Hong Kong China; 5 Department of Social Work, Chinese University of Hong Kong, Shatin, New Territories, Hong Kong China; 6 School of Social Sciences, Monash University, 20 Chancellors Walk, Melbourne, 3800 Australia; 7 Department of Occupational Therapy, Shu-Zen Junior College of Medicine and Management, 452, Huanqiu Rd., Luzhu Dist, Kaohsiung, 821004 Taiwan; 8 Division of Colon and Rectal Surgery, Department of Surgery, E-Da Hospital, School of Medicine, College of Medicine, I-Shou University, Kaohsiung, 824 Taiwan; 9 Chinese Academy of Education Big Data, Qufu Normal University, 57 Jingxuan West Rd, Qufu, 273165 China; 10 Department of Healthcare Administration, Asia University, Taichung, 41354 Taiwan; 11 Department of Family Medicine, Asia University Hospital, Taichung, 41354 Taiwan; 12 Department of Kinesiology, Health, and Leisure, Chienkuo Technology University, Changhua, 50094 Taiwan; 13 International Gaming Research Unit, Psychology Department, Nottingham Trent University, Nottingham, UK; 14 University of Religions and Denominations, Qom, Iran; 15 Department of Public Health, College of Medicine, National Cheng Kung University, No. 1, University Rd., East Dist, Tainan, 701401 Taiwan; 16 Biostatistics Consulting Center, National Cheng Kung University Hospital, College of Medicine, National Cheng Kung University, No. 1, University Rd., East Dist, Tainan, 701401 Taiwan; 17 INTI International University, Negeri Sembilan, Nilai, Malaysia

**Keywords:** Tendency to avoid physical activity and sport scale, Differential item functioning, Physical activity, Rasch, China, Young adults

## Abstract

**Background:**

The benefits of physical activity are well-known to prevent multiple long-term health conditions. Physical appearance and weight-related stigma may influence individuals’ decision to engage in physical activity and sport. Therefore, the present study examined the psychometric properties of a newly developed instrument, the Tendency to Avoid Physical Activity and Sport Scale (TAPAS), using modern test theory.

**Methods:**

A total of 2319 university students were recruited from mainland China and they completed the TAPAS. Rasch analysis was used to examine the TAPAS’ rating scaling functioning, test unidimensionality, item hierarchy, ceiling and floor effects, and differential item functioning (DIF). Moreover, the concurrent validity of the TAPAS was examined using the Weight Self-Stigma Questionnaire (WSSQ), Weight Bias Internalization Scale (WBIS), and body mass index (BMI).

**Results:**

Unidimensionality was confirmed except for one item. Items corresponding to attitude toward physical activity were more easily adopted compared to items corresponding to actual behavioral aspects. No ceiling and floor effects were found. No DIF existed in the TAPAS items. The TAPAS was strongly correlated with both the WSSQ and WBIS, but not BMI.

**Conclusion:**

The study showed that overall, the TAPAS has robust psychometric properties. However, future research needs to address the misfit item and explore the feasibility of applying the TAPAS to other populations including wider ethnic groups, age ranges, and life stages.

**Supplementary Information:**

The online version contains supplementary material available at 10.1186/s40359-023-01377-y.

## Introduction

Insufficient physical activity has been found to be one of the leading risk factors for non-communicable, long-term health conditions, such as heart disease [1], cancer [2, 3], chronic respiratory disease [4, 5], and diabetes mellitus [6]. In addition, prior systematic reviews have shown that physical activity and sports have positive and significant effects on individuals’ wellbeing and quality of life [7, 8]. The World Health Organization (WHO) has provided clear suggestions and guidelines regarding the levels of physical activity for different age groups. For example, adults aged 18 to 64 years old should perform at least 150 min of moderate-intensity aerobic physical activity, or at least 75 min of vigorous-intensity aerobic physical activity (or an equivalent combination) per week to maintain good health [9]. Although the benefits of physical activity are well-known and have been promoted over the decades, studies still show that approximately one-third of adults worldwide do not reach the recommended levels of physical activity [10, 11].

Several studies have reported the pragmatic barriers that prohibit individuals in participating in physical activity, such as lack of childcare support, and lack of financial and community resources [12], as well as lack of social support and motivation [13]. A recent study proposed and confirmed that participants’ concerns about their own physical appearance and weight-related stigma may influence their enjoyment when participating in physical activity [14]. More specifically, the researchers assessed enjoyment in participation (using the Physical Activity Enjoyment Scale), weight stigma (using five-item Perception of Teasing Scale and Weight Bias Internalization Scale), and avoidance participation (using the TAPAS). They found that decreased enjoyment and stigmatized perceptions could potentially lead to individuals deciding to avoid engaging in physical activity [14]. Prior findings have also indicated that body appearance evaluation is strongly associated with physical activity levels among adolescents [15] and university students [16]. Therefore, it is of great importance to evaluate individuals’ physical appearance and weight stigma concerns because such psychosocial aspects might help to answer the unresolved issues on physical activity participation.

Most previous studies have evaluated the tendency of avoiding physical activity with simply one or two items [17]. Until recently, there had been no validated psychometric instrument developed which could be practically used in clinical or community settings. Therefore, Bevan et al. [18] developed the Tendency to Avoid Physical Activity and Sport Scale (TAPAS) using rigorous assessment development guidelines. Moreover, the development of the TAPAS is supported by the cyclic obesity/weight-based stigma (COBWEBS) framework [19]. More specifically, the COBWEBS framework proposes that weight-related self-stigma is a trigger for an individual to engage in behaviors that cause weight problems (e.g., eating disorders and physical activity avoidance). The TAPAS assesses physical activity and exercise avoidance that could be due to individuals’ weight-related self-stigma and fits within this framework. Their goal was to create a feasible psychometric instrument which would allow clinicians and researchers to better understand the role of weight and appearance-related concerns in physical activity participation [18].

The TAPAS was first tested in an English-speaking (Australian) undergraduate student population and showed the initial evidence and feasibility [14]. Assessment evaluation is a fundamental process to ensure the reliability and validity of any newly developed instruments [20]. More specifically, how each item’s content reflects the purpose of the instrument, the scale construction, and test dimensionality should be examined to ensure the items in the TAPAS have an appropriate level of efficacy in evaluating the construct it claims to assess. To the best of the present authors’ knowledge, the TAPAS has not been psychometrically evaluated using advanced techniques (i.e., Rasch analysis). Therefore, the present study used the Rasch measurement model to further examine the psychometric properties of the TAPAS. Rasch models adopt probabilistic test theory and its precise calibration for each scale item is beneficial for individual test item examination and overall optimizing the test [21]. Due to its strength and usefulness, it has been used extensively in assessment development in a variety of fields [22]. The present study addresses four specific research questions: (i) How does the rating scale function in the TAPAS? (RQ1), (ii) Do all of the 10 items included in TAPAS demonstrate unidimensionality? (RQ2), (iii) Do ceiling or floor effects exist in the TAPAS? (RQ3), and (iv) Do any of the 10 TAPAS items display differential item functioning (DIF) across gender? (RQ4).

## Methods

### Participants

After obtaining the approval from the Institutional Review Board of Jiangxi Psychological Consultant Association (IRB ref: JXSXL-2021‐J99), the present authors sent an invitation to 25 universities in mainland China to seek their assistance in data collection. Of these, 19 universities across 13 provinces in mainland China agreed to assist in distributing the survey link. Convenience sampling was conducted using an online survey between August and October 2022 via an online hyperlink or a QR code. The eligibility criteria for participant inclusion were being: (i) an adult (i.e., aged 18 years or above); (ii) a university student in mainland China; and (iii) willing to provide electronic consent for participation.

### Translation procedures for TAPAS

As aforementioned, the TAPAS is a newly developed scale developed with an English-speaking population (Australians). Therefore, the present authors used a standard translation procedure to translate the English version of the TAPAS to a Chinese version of the TAPAS [23]. In the first stage of the translation procedure, two bilingual translators who were native Chinese (Mandarin) speakers carried out a forward translation independently. In the second stage, the two translators worked with one of the present authors (C-YL) to reconcile a Chinese TAPAS for back translation. In the third stage, a third bilingual translator who was also a native Chinese speaker and was not aware of the English TAPAS translated the reconciled Chinese TAPAS into English. In the fourth stage, one of the present authors (C-YL) called an expert panel meeting with the composition of all translators and various experts (with expertise in pediatrics, public health, psychometrics, physical activity, and weight) to finalize the Chinese TAPAS with the use of all the TAPAS translations and the original TAPAS. In the final stage, the finalized Chinese TAPAS was sent out to several university students to evaluate the readability, and the Chinese TAPAS was revised with some minor word changes.

### Measures^1^

#### Tendency to Avoid Physical Activity and Sport Scale (TAPAS)

The Tendency to Avoid Physical Activity and Sport Scale [18] has a total of 10 items with five items evaluating physical activity and five items evaluating sport contexts. Items are rated on a five-point scale from 1 (*strongly disagree*) to 5 (*strongly agree*), with higher scores indicating greater tendency to avoid physical activity or sport. The TAPAS assesses physical activity and sport avoidance associated with weight-related self-stigma. Moreover, the TAPAS has been validated among undergraduate students [14]. The internal consistency of the original TAPAS was excellent (Cronbach’s α = 0.95) [14].

#### Weight self-stigma questionnaire (WSSQ)

The Weight Self-Stigma Questionnaire is a commonly used instrument that assesses weight-related self-stigma. It has two subscales that assess self-devaluation and fear of enacted stigma, respectively [24–26]. Each subscale has six items that are rated on a five-point Likert scale from 1 (*strongly disagree*) to 5 (*strongly agree*). A higher score indicates a higher level of weight-related self-stigma. The Chinese version of the WSSQ was used in the present study which has been shown to have satisfactory psychometric properties [27]. The internal consistency of the original WSSQ was very good (Cronbach’s α = 0.88) [25]. The Cronbach’s α of the WSSQ in the present study was 0.90.

#### Weight bias internalization scale (WBIS)

The Weight Bias Internalization Scale is a widely used instrument that assesses how an individual accepts weight-based stereotypes [28]. It has 11 items that are self-rated on a five-point Likert scale from 1 (*strongly disagree*) to 5 (*strongly agree*) [29]. When an individual receives a higher score, it indicates a higher level of weight-related self-stigma. The Chinese version of the WBIS was used in the present study and it has been shown to have satisfactory psychometric properties [30]. The internal consistency of the original WBIS was excellent (Cronbach’s α = 0.90) [29]. The Cronbach’s α of the WBIS in the present study was 0.96.

### Data analysis

In the present study, Rasch analysis was used to examine the TAPAS’ rating scaling function, test unidimensionality (i.e., to see if all items assessed the same latent construct in the TAPAS), item hierarchy, ceiling and floor effects, and differential item functioning (DIF) across male and female participants. A previous factor analysis study confirmed the single-factor solution for the TAPAS [18]. Therefore, all 10 items were considered to be assessing the same latent construct in the TAPAS (i.e., physical activity and sport avoidance) for Rasch analysis.

The partial credit model [31] was applied in the Rasch analysis to allow individual calibration for the TAPAS items that might have different rating scales. Additionally, it was investigated whether the five-point Likert scale had sufficient participants (i.e., at least 10) in each rating category as well as whether each rating category advanced monotonically throughout the five-point Likert scale levels.

The test unidimensionality of the TAPAS was examined with the goodness-of-fit statistics derived from the Rasch analysis. Two type of mean-squares (MnSq) statistics were used because they showed the amount of distortion of the underlying test structure of the TAPAS. The outfit MnSq is more sensitive to unexpected outliers. It was expected to be less than 2 in all TAPAS items. The infit MnSq is more sensitive to on-target observations with the expected value of 1.0. Infit MnSq statistics between 0.5 and 1.5 associated with a standardized mean square scored (Zstd) between − 2 to + 2 are desired [32]. If any TAPAS item demonstrated an infit MnSq > 1.5, it is considered a threat to TAPAS’s validity because it may suggest that the items are unpredictable with unmodeled noise [33]. If all the TAPAS items have satisfactory infit MnSq statistics, then the unidimensionality of the test can be supported. Furthermore, the item calibrations derived from the Rasch analysis were used to examine the hierarchy of the TAPAS items.

Additionally, the ceiling and floor effects were examined to see if any participants with extreme scores were outliers, which may affect the reliability of the TAPAS. It was expected that there should be less than 15% of the total participants that would achieve the maximum or the minimum possible scores in the TAPAS to be considered as having no ceiling or floor effects, respectively [34].

Moreover, differential item functioning (DIF) analysis was carried out to investigate the interaction between gender that might cause underlying bias. It is generally acceptable to have less than 5% of items that display DIF to support unidimensionality [35–37]. The Rasch-Welch *t*-statistic was utilized to identify any item with statistically significant DIF (*p* < .05). DIF contrasts (between male and female participants) less than 0.5 logit are negligible while the contrasts between 0.5 and 1 logit are considered moderate. Contrasts over 1 are defined as substantial [38]. Moreover, according to the Rasch guidelines on the required sample size, each rating category should have a minimum of at least of 10 participants to generate stable estimates [40]. Winsteps version 5.3.3.0 was used to perform the Rasch analysis. Other demographic and descriptive statistics were conducted with IBM SPSS version 28.0.

The concurrent validity for the TAPAS was examined using three external measures: body mass index (BMI), Weight Self-Stigma Questionnaire (WSSQ) and the Weight Bias Internalization Scale (WBIS). Spearman correlation coefficient was used with the criteria that 0.1 to 0.3 = weak correlation; 0.4 to 0.7 = moderate correlation; > 0.7 = strong correlation [39]. Lastly, internal consistency of the TAPAS was examined for all the 10 items using Cronbach’s α.

## Results

All participants who reported extreme values for their BMI (below 10 or over 50) and those reporting identical answers to all the questions were removed during data cleaning. Therefore, the final sample comprised 2319 higher education students from mainland China. The majority of them were university students (n = 1872, 80.7%), single (n = 2263, 97.6%), with a mean age of 20.16 years (SD = 1.75), and approximately 57% were females. Students were recruited from diverse disciplines with the top five majors being science (27.2%), engineering (21.6%), education (12.2%), management (11.6%), and art (9.0%). Further demographic details can be found in Table 1.

**Table 1:** Participants’ demographics (N = 2319)

	Mean ± SD	n (%)
**Age**	20.16 ± 1.75	
**Gender**		
Male		992 (42.8)
Female		1327 (57.2)
**Education level**		
University		1872 (80.7)
Associate^1^		447 (19.3)
**Department/Major**		
Philosophy		19 (0.8)
Economics		185 (8.0)
Law		38 (1.6)
Education		284 (12.2)
Literature		85 (3.7)
History		3 (0.1)
Sciences		631 (27.2)
Engineering		502 (21.6)
Agriculture		14 (0.6)
Medical		31 (1.3)
Military		7 (0.3)
Management		270 (11.6)
Art		208 (9.0)
Interdisciplinary		42 (1.8)
**Only child**		
No		1821 (78.5)
Yes		498 (21.5)
**Marriage status**		
Single		2263 (97.6)
Married		21 (0.9)
Divorced		18 (0.8)
Separated		17 (0.7)

^1^ An associate degree is an undergraduate degree which is awarded after completing a short course in university or college (usually lasting 2 to 3 years)

The Rasch analysis showed that the TAPAS had sufficient participants (i.e., over 10) to generate stable estimates for each rating category. Additionally, all 10 items had average measures that advanced monotonically (i.e., the difficulty of each category in the responses increased by order; scoring 1 is easier than scoring 2 and scoring 2 is easier than scoring 3, etc.) using the five-point Likert scale. The monotonical advancement was assessed using the average rating measures (Table 2). Regarding the item fitness statistics, all the test items had outfit MnSq less than the cut-off criteria of 2 along with the desired infit MnSq. However, Item 10 (*I would prefer to participate in physical activity in a more private setting*) demonstrated infit MnSq misfit with the Zstd above 2 (Table 2), which indicated a misfit in the Rasch model’s expectation. Therefore, this misfitting item was removed and the partial credit Rasch model was rerun with the remaining nine items in the TAPAS. The results (Table 3) showed that all the remaining nine items fitted the Rasch model with acceptable values of infit and outfit MnSq and Zstd. Moreover, the average measures across rating categories advanced monotonically. These findings confirmed the unidimensionality of the nine-item TAPAS and supported the construct validity of the TAPAS. When examining the item hierarchy, the most difficult item (i.e., an item that requires a relatively high trait level to be answered correctly) with the TAPAS was Item 1 (*I find myself avoiding participating in sport because of my weight*) and the easiest item (i.e., an item that requires a relatively low trait level to be answered correctly) was the Item 5 (*I am concerned about what other people think of my appearance when I participate in sport*). Table 3 contains the detailed item calibrations.

**Table 2:** Rasch analyses of the 10 TAPAS items

Item	Measure	SE	Infit		Outfit		Average Rating Measure^1^				
			*MnSq*	*Zstd*	*MnSq*	*Zstd*	1	2	3	4	5
1. I find myself avoiding participating in sport because of my weight.	0.50	0.04	1.41	9.90	1.48	9.90	-5.11	-1.86	− 0.07	1.29	3.50
2. I avoid participating in sport because of my fear of being judged about my lack of physical ability.	0.21	0.04	1.07	1.99	1.09	2.51	-5.39	-1.97	− 0.07	1.17	3.56
3. I worry about participating in sport because I don’t like how my body looks when playing sport.	0.15	0.04	0.85	-4.71	0.84	-4.79	-5.60	-1.97	− 0.01	1.20	3.59
4. I am afraid other people will notice my physical flaws when I participate in sport.	− 0.25	0.04	0.86	-4.45	0.86	-4.41	-5.93	-2.18	− 0.27	0.64	3.46
5. I am concerned about what other people think of my appearance when I participate in sport.	− 0.44	0.04	1.11	3.28	1.12	3.61	-6.19	-2.27	− 0.49	0.56	3.03
6. I avoid physical activity because I might get teased about my weight.	0.35	0.04	0.80	-6.51	0.76	-7.49	-5.50	-1.85	0.11	1.49	4.17
7. I avoid physical activity because of my fear of being judged about my physical appearance.	0.15	0.04	0.66	-9.90	0.65	-9.90	-5.77	-1.97	0.01	1.36	4.02
8. I avoid physical activity because I worry that people may make negative comments about my body.	0.25	0.04	0.65	-9.90	0.64	-9.90	-5.62	-1.97	0.09	1.42	4.27
9. I avoid physical activity because I worry people may be thinking negatively about my physical appearance.	0.18	0.04	0.71	-9.80	0.71	-9.48	-5.68	-1.97	0.01	1.40	4.08
10. I would prefer to participate in physical activity in a more private setting.^+^	-1.09	0.04	1.80	9.90	1.87	9.90	-6.36	-2.39	− 0.95	− 0.12	1.77

**Note.**^1^The average measure is expected to increase with category value [33]. ^+^Infit statistics > 1.5 associated with Zstd > 2: item misfit. MnSq = Mean Square; Zstd = Standardized score

**Table 3:** Rasch analyses of the nine TAPAS items

Item	Measure	SE	Infit		Outfit		Average Rating Measure^1^				
			*MnSq*	*Zstd*	*MnSq*	*Zstd*	1	2	3	4	5
1. I find myself avoiding participating in sport because of my weight.	0.41	0.04	1.50	9.90	1.57	9.90	-5.74	-2.11	− 0.08	1.41	3.70
2. I avoid participating in sport because of my fear of being judged about my lack of physical ability.	0.10	0.04	1.13	3.65	1.14	3.93	-6.05	-2.25	− 0.07	1.23	3.74
3. I worry about participating in sport because I don’t like how my body looks when playing sport.	0.03	0.04	0.92	-2.50	0.90	-2.86	-6.27	-2.25	− 0.01	1.26	3.75
4. I am afraid other people will notice my physical flaws when I participate in sport.	− 0.41	0.04	1.01	0.37	1.02	0.53	-6.61	-2.47	− 0.32	0.58	3.54
5. I am concerned about what other people think of my appearance when I participate in sport.	− 0.64	0.04	1.33	9.11	1.36	9.90	-6.83	-2.60	− 0.57	0.46	3.06
6. I avoid physical activity because I might get teased about my weight.	0.25	0.04	0.83	-5.31	0.78	-6.53	-6.17	-2.13	0.14	1.61	4.32
7. I avoid physical activity because of my fear of being judged about my physical appearance.	0.04	0.04	0.71	-9.34	0.70	-9.77	-6.45	-2.25	0.01	1.44	4.16
8. I avoid physical activity because I worry that people may make negative comments about my body.	0.15	0.04	0.70	-9.76	0.69	-9.90	-6.29	-2.26	0.11	1.49	4.42
9. I avoid physical activity because I worry people may be thinking negatively about my physical appearance.	0.07	0.04	0.79	-6.69	0.80	-6.25	-6.34	-2.25	0.01	1.45	4.24

**Note.**^1^The average measure is expected to increase with category value [33]. ^+^Infit statistics > 1.5 associated with Zstd > 2: item misfit. MnSq = Mean Square; Zstd = Standardized score

When examining the response pattern of the participants, it was found that 16 (0.7%) and 302 (13%) out of the 2319 students had achieved the maximum and the minimum TAPAS scores, respectively. Therefore, there were no ceiling or floor effects in the TAPAS, which also confirmed that TAPAS items were well targeted to the current population group. Additionally, no significant DIF in any of the TAPAS items were found relative to gender (Table 4). All the DIF contrasts between male and female participants were between − 0.26 and 0.30 logit, which were within the negligible range.

**Table 4:** Gender DIF for the nine TAPAS items

Item	Male Measure	Female Measure	*t*	*p*	DIF Contrast*
1. I find myself avoiding participating in sport because of my weight.	0.46	0.49	− 0.45	0.65	− 0.04
2. I avoid participating in sport because of my fear of being judged about my lack of physical ability.	0.17	0.29	-1.53	0.13	− 0.12
3. I worry about participating in sport because I don’t like how my body looks when playing sport.	0.20	0.16	0.43	0.67	0.03
4. I am afraid other people will notice my physical flaws when I participate in sport.	− 0.14	− 0.44	3.96	< 0.001	0.30
5. I am concerned about what other people think of my appearance when I participate in sport.	− 0.50	− 0.52	0.28	0.78	0.02
6. I avoid physical activity because I might get teased about my weight.	0.26	0.52	-3.36	< 0.001	− 0.26
7. I avoid physical activity because of my fear of being judged about my physical appearance.	0.11	0.23	-1.54	0.12	− 0.12
8. I avoid physical activity because I worry that people may make negative comments about my body.	0.23	0.32	-1.17	0.24	− 0.09
9. I avoid physical activity because I worry people may be thinking negatively about my physical appearance.	0.13	0.30	-2.21	0.03	− 0.17

**Note.** *Positive values indicate that the probability of the item endorsement is easier for male participants than female participants. DIF = Differential Item Functioning

The correlation between TAPAS and the other three external measures were investigated. The Spearman correlation indicated strong correlations between TAPAS and WSSQ (*r* = .71, *p* < .001) and WBIS (*r* = .76, *p* < .001). No significant correlation was found between the TAPAS and BMI (Table 5). Lastly, the TAPAS showed excellent internal consistency (Cronbach’s α = 0.96).

**Table 5:** Spearman’s correlation between TAPAS and external measurements

	TAPAS	BMI	WSSQ	WBIS
TAPAS	1.000	0.054	0.713**	0.757**
BMI		1.000	0.086**	0.096**
WSSQ			1.000	0.856**
WBIS				1.000

Note. **p < .001; 0.1 to 0.3 = weak correlation; 0.4 to 0.7 = moderate correlation; > 0.7 = strong correlation

TAPAS = Tendency to Avoid Physical Activity and Sport Scale; BMI = body mass index; WSSQ = Weight Self-Stigma Questionnaires; WBIS = Weight Bias Internalization Scale

## Discussion

To the best of the authors’ knowledge, the present large-scale study of university students across mainland China is the first study to examine the psychometric properties of the Tendency to Avoid Physical Activity and Sport Scale (TAPAS). The study applied a widely used rigorous analysis incorporating modern test theory (i.e., Rasch analysis) [21, 40] to examine the psychometric properties of the newly developed TAPAS to evaluate participants’ tendency to avoid physical activity and sport due to appearance or weight-stigma concerns. The results successfully provided evidence of the TAPAS’ construct validity and confirmed the unidimensionality of the test items in the nine-item version. Also, no ceiling or floor effects were found. Moreover, no DIF was found across male and female populations. Therefore, the TAPAS is a reliable and valid assessment tool that can be used to assess psychosocial concerns regarding appearance and weight-stigma in relation to avoiding physical activity and sport.

For the rating scale functioning, each rating category in the TAPAS substantively defined to represent a higher level of tendency to avoid physical activity and sport. The results showed that all items had average measures that advanced monotonically with the designed Likert scale, which confirmed that the five-point rating scale functioned well in capturing the underlying construct (i.e., tendency to avoid physical activity and sport). In other words, individuals with a lower tendency to engage in physical activity and sport rated lower on the TAPAS compared to individuals with a higher tendency.

When examining the TAPAS item structure, Item 10 (*I would prefer to participate in physical activity in a more private setting*) showed misfit MnSq with significant Zstd, which indicated that this particular item may be evaluating a different underlying trait that was not intended to be assessed [32]. Previous studies have found that individuals with obesity or weight concerns may exclude themselves from specific exercise settings [41, 42]. This item was originally designed to assess whether individuals would avoid engaging in physical activity in a public, open-space workout setting due to their appearance/weight concerns. However, when closely examining this item, the authors noticed that the term *“private setting”* stood out. The authors were of the view that this could be a misleading term which could imply a specific setting that only an individual with special privileges or being of high socioeconomic status have access to (e.g., the VIP room in a gym or a reserved room in a community sport center). Therefore, this misfitting item in the Rasch model might have been mis-interpreted and rated differently among the study participants. Consequently, this item was removed from the subsequent analysis. Future studies should consider either revising the item description or further examining whether this item should be retained in the TAPAS.

The item measures derived from the Rasch analysis helped to depict the item hierarchy along the linear construct to express the participants’ tendency to avoid physical activity and sport. The most easily adopted item was Item 5 (*I am concerned about what other people think of my appearance when I participate in sport*) followed by the Item 4 (*I am afraid other people will notice my physical flaws when I participate in sport*). The most difficult adopted item was Item 1 (*I find myself avoiding participating in sport because of my weight*) followed by the Item 6 (*I avoid physical activity because I might get teased about my weight*). Previous studies have demonstrated that attitudes toward physical activity are the most easily adopted variable and are consistently rated as a significant predictor of physical activity intentions among older adults [43] and students [44]. Item 4 and Item 5 in the TAPAS more concern a person’s attitude about engaging in physical activity, which align with prior findings that individuals’ attitude significantly associated with their intention to engage in physical activity. In contrast, Item 1 and item 6 in the TAPAS more concern the actual behaviors of avoiding physical activity. Pachankis [45] illustrated the psychological stages of how individuals process stigma conditions. Weight-related stigma may result in negative effects and attitude. Subsequently, such negative effects can lead to self-stigmatizing behaviors, such as avoidance or withdrawal from specific behaviors or social interactions [46]. Therefore, the current item hierarchy results were consistent with the prior findings that behavior-relevant items are expected to be the items least likely to be adopted by individuals.

Previous research shows that gender plays a critical role on how individuals perceive their body image [47]. More specifically, although both males and females have concerns regarding their body images, the way it manifests may be quite different (e.g., females feeling pressure to be thinner, and males feeling pressure to be more muscular). One possible explanation regarding no DIF being found in the TAPAS between gender could the “neutral phrasing” in the TAPAS. That is, no descriptions are prone to specific body image concerns of males or females in the TAPAS. Therefore, it can be used consistently with both male and female populations. Multiple surveillance studies which have investigated physical activity levels worldwide show that females have lower physical activity participation in comparison to males [11, 48]. Moreover, females have been found to be significantly more dissatisfied with their bodies than males [47, 49]. Therefore, it is critical that the newly developed TAPAS can be used validly and equitably for both males and females.

Finally, strong and significant associations were found between the TAPAS and the WSSQ, and the TAPAS and the WBIS. These findings are consistent with previous studies where stigma experiences were highly correlated with an increased desire to avoid exercise [14, 50]. These results confirmed the concurrent validity of the TAPAS. The associations between these standardized instruments were expected because they either evaluate the level of weight-related self-stigma or how it is being internalized to the individual’s belief, which are all closely related to their tendency to avoid physical activity and sport due to weight-related concerns. Notably, the TAPAS was not associated with BMI, which means, participants’ body fatness level was not directly relevant to their perspectives of physical activity and sport participation. A prior study indicated that individuals can experience and be aware of weight-related stigma. However, they do not necessarily apply the prejudice to themselves nor accept and internalize the stigma [51]. Therefore, in this case, it does not influence their tendency to take in part of any physical activity or sports. This may explain why physical activity and sport avoidance tendency was not directly related to BMI.

### Limitations and future research recommendations

Although the TAPAS was designed to be used across a variety of populations [14, 52], the present study (while large-scale) was a convenience sample that only comprised university students. Therefore, future studies should explore the feasibility of the TAPAS to other population groups with more representative samples. One item (Item 10: *I would prefer to participate in physical activity in a more private setting*) did not fit the Rasch model. This needs to be further examined in future studies to ensure the item wording does not deviate from the main purpose of the item. Additionally, because individuals change their attitude toward how they view themselves and how they handle others’ judgment, future studies should be conducted to examine potential longitudinal effects. A prior study showed that body dissatisfaction and self-esteem differ across ethnic groups [53], and how individuals perceive their body image varies across different ages [47]. Therefore, further studies are suggested to include individuals from different ethnic groups and from varied life stages. In addition, the present study did not measure actual level of physical activity engagement. It is therefore unclear if the TAPAS and its items actually link to low levels of physical activity engagement. Consequently, future studies are needed to collect actual physical activity data to revalidate the TAPAS. Moreover, the present study did not collect the information as to whether any of the participants were athletes. Given that physical activity is the core behavior of athletes, it is important to know if the TAPAS can be used among athletes. Therefore, future studies may want to examine the psychometric properties of the TAPAS among athlete populations. Last, use of self-report data in relation to a sensitive topic, such as providing height and weight (to calculate BMI), could potentially contribute to self-reporting bias and social desirability bias. Future studies should consider adding objective measurements to increase data validity.

## Conclusion

The present study applied Rasch analysis to examine the psychometric properties of the newly developed TAPAS among a university students’ population in mainland China. The study findings suggest that the TAPAS is a valid and reliable self-report psychometric instrument. Using this scale, weight and appearance concerns can be evaluated and then addressed with appropriate interventions to promote greater physical activity participation.

### Electronic supplementary material

Below is the link to the electronic supplementary material.

Supplementary Material 1

## Data Availability

The datasets used and/or analysed during the current study are available from the corresponding author on reasonable request.

## Footnotes

1 ***please see supplementary file for the Chinese versions of these measures***

## List of abbreviations

BMI: body mass index
DIF: differential item functioning
MnSq: Mean-square
Zstd: standardized score
TAPAS: Tendency to Avoid Physical Activity and Sport Scale
WBIS: Weight Bias Internalization Scale
WSSQ: Weight Self-Stigma Questionnaire
