# Correction: The Tendency to Avoid Physical Activity and Sport Scale (TAPAS): Rasch analysis with differential item functioning testing among a Chinese sample

**DOI:** 10.1186/s40359-023-01451-5

**Published:** 2023-11-30

**Authors:** Chia-Wei Fan, Yen-Ling Chang, Po-Ching Huang, Xavier C. C. Fung, Ji-Kang Chen, Nadia Bevan, Kerry S. O’Brien, Ya-Chin Yeh, Hsin-Pao Chen, I.-Hua Chen, I.-Ching Lin, Mark D. Griffiths, Chung-Ying Lin

**Affiliations:** 1 Department of Occupational Therapy, AdventHealth University, 671 Winyah Drive, Orlando, FL 32803 USA; 2 Department of Family Medicine, Cardinal Tien Hospital, No. 362, Zhongzheng Rd., Xindian Dist New Taipei, 231403 Taiwan; 3 Institute of Allied Health Sciences, College of Medicine, National Cheng Kung University, University Rd., No. 1, East Dist Tainan, 701401 Taiwan; 4 Department of Rehabilitation Sciences, Faculty of Health and Social Sciences, The Hong Kong Polytechnic University, 11 Yuk Choi Rd., Hung Hom, Kowloon, Hong Kong China; 5 Department of Social Work, Chinese University of Hong Kong, Shatin, New Territories, Hong Kong China; 6 School of Social Sciences, Monash University, 20 Chancellors Walk, Melbourne, 3800 Australia; 7 Department of Occupational Therapy, Shu-Zen Junior College of Medicine and Management, 452, Huanqiu Rd., Luzhu Dist Kaohsiung, 821004 Taiwan; 8 Division of Colon and Rectal Surgery, Department of Surgery, E-Da Hospital, School of Medicine, College of Medicine, I-Shou University, Kaohsiung, 824 Taiwan; 9 Chinese Academy of Education Big Data, Qufu Normal University, 57 Jingxuan West Rd, Qufu, 273165 China; 10 Department of Healthcare Administration, Asia University, Taichung, 41354 Taiwan; 11 Department of Family Medicine, Asia University Hospital, Taichung, 41354 Taiwan; 12 Department of Kinesiology, Health, and Leisure, Chienkuo Technology University, Changhua, 50094 Taiwan; 13 International Gaming Research Unit, Psychology Department, Nottingham Trent University, Nottingham, UK; 14 University of Religions and Denominations, Qom, Iran; 15 Department of Public Health, College of Medicine, National Cheng Kung University, No. 1, University Rd., East Dist, Tainan, 701401 Taiwan; 16 Biostatistics Consulting Center, National Cheng Kung University Hospital, College of Medicine, National Cheng Kung University, No. 1, University Rd., East Dist Tainan, 701401 Taiwan; 17 INTI International University, Nilai, Negeri Sembilan Malaysia

**Correction: BMC Psychology 11, 369 (2023)**

**https://doi.org/10.1186/s40359-023-01377-y**

Following publication of the original article [1], the authors flagged several errors: the title had not been capitalized correctly; affiliations 8, 14, and 15 were incorrectly detailed; the funding declaration was complete ('E-Da Hospital (EDAHP109027' had not been acknowledged). These errors have since been corrected in the published article. The authors thank you for reading this erratum and apologize for any inconvenience caused.
