# Supplementary material for: The Tendency to Avoid Physical Activity and Sport Scale (TAPAS): Rasch analysis with differential item functioning testing among a Chinese sample
Source: BMC Psychol. 2023 Nov 4;11:369. doi: 10.1186/s40359-023-01377-y (PMC10625701; doi:10.1186/s40359-023-01377-y)
Supplement: Supplementary file 1 — Supplementary Material 1 [file 40359_2023_1377_MOESM1_ESM.docx]

Supplementary File. Chinese versions of the three measures used in the present study.

*Tendency to Avoid Physical Activity and Sport (TAPAS) scale:*

| Scale item | Strongly disagree  非常不同意 | Disagree  不同意 | Neutral  中立 | Agree  同意 | Strongly agree  非常同意 |
| --- | --- | --- | --- | --- | --- |
| I find myself avoiding participating in sport because of my weight  我会因为体重因素而避免参与运动 |  |  |  |  |  |
| I avoid participating in sport because of my fear of being judged about my lack of physical ability  我因为害怕被批评体能不足而避免参与运动 |  |  |  |  |  |
| I worry about participating in sport because I don’t like how my body looks when playing sport  我对参与运动比较担忧，因为我不喜欢运动时身体看起来的样子 |  |  |  |  |  |
| I am afraid other people will notice my physical flaws when I participate in sport  当我运动时，我害怕其他人注意到我身体的缺点 |  |  |  |  |  |
| I am concerned about what other people think of my appearance when I participate in sport  当我运动时，我很在意其他人如何看待我的外表 |  |  |  |  |  |
| I avoid physical activity because I might get teased about my weight  我因为可能会被人嘲笑体重，而避免身体活动 |  |  |  |  |  |
| I avoid physical activity because of my fear of being judged about my physical appearance  我因为害怕被人评论外表，而避免身体活动 |  |  |  |  |  |
| I avoid physical activity because I worry that people may make negative comments about my body  我因为担心他人对我的身材有负面评论，而避免身体活动 |  |  |  |  |  |
| I avoid physical activity because I worry people may be thinking negatively about my physical appearance  我因为担心人们对我的外表有负面看法，而避免身体活动 |  |  |  |  |  |
| I would prefer to participate in physical activity in a more private setting  我会比较喜欢在更隐私的场所从事身体活动 |  |  |  |  |  |

*Weight Bias Internalization Scale (WBIS):*

|  | 非常不同意  Strongly disagree | 不同意  Disagree | 中立  Neutral | 同意  Agree | 非常同意  Strongly agree |
| --- | --- | --- | --- | --- | --- |
| 1.我的体重让我觉得自己与其他人一样能干  As an overweight person, I feel that I am just as competent as anyone | 1 | 2 | 3 | 4 | 5 |
| 2.因为我的体重，我比别人欠缺吸引力  I am less attractive than most other people because of my weight | 1 | 2 | 3 | 4 | 5 |
| 3.我会因别人的想法而对我的体重感到不安  I feel anxious about being overweight because of what people might think of me | 1 | 2 | 3 | 4 | 5 |
| 4.我希望能大幅改变自己的体重  I wish I could drastically change my weight | 1 | 2 | 3 | 4 | 5 |
| 5.每当我想到自己的体重时，我会感到心情不好  Whenever I think a lot about being overweight, I feel depressed | 1 | 2 | 3 | 4 | 5 |
| 6.我讨厌我的体重  I hate myself for being overweight | 1 | 2 | 3 | 4 | 5 |
| 7.我主要用体重去判断我作为一个人的价值  My weight is a major way that I judge my value as a person | 1 | 2 | 3 | 4 | 5 |
| 8.因为我的体重，我不认为我值得拥有真正美满的社交生活  I don't feel that I deserve to have a really fulfilling social life, as long as I'm overweight | 1 | 2 | 3 | 4 | 5 |
| 9.我能接受现在的体重  I am OK being the weight that I am | 1 | 2 | 3 | 4 | 5 |
| 10.因为我的体重，我不能感受到真正的自我  Because I'm overweight, I don't feel like my true self | 1 | 2 | 3 | 4 | 5 |
| 11.因为我的体重，我不认为会有吸引力的人想和我交朋友  Because of my weight, I don't understand how anyone attractive would want to date me | 1 | 2 | 3 | 4 | 5 |

*Weigh Self-Stigma Questionnaire (WSSQ):*

|  | 非常不同意  Strongly disagree | 不同意  Disagree | 中立  Neutral | 同意  Agree | 非常同意  Strongly agree |
| --- | --- | --- | --- | --- | --- |
| 1.我一定会不断变回过重的状态。  I’ll always go back to being overweight | 1 | 2 | 3 | 4 | 5 |
| 2.我引起了自己的体重问题  I caused my weight problems | 1 | 2 | 3 | 4 | 5 |
| 3.我因自己的体重问题而感到内疚  I feel guilty because of my weight problems | 1 | 2 | 3 | 4 | 5 |
| 4.我过重的原因是因为我是个软弱的人  I became overweight because I’m a weak person | 1 | 2 | 3 | 4 | 5 |
| 5.如果我坚强些，我永远不会有任何与体重有关的问题  I would never have any problems with weight if I were stronger | 1 | 2 | 3 | 4 | 5 |
| 6.我没有足够的自我控制能力去维持健康的体重  I don’t have enough self-control to maintain a healthy weight | 1 | 2 | 3 | 4 | 5 |
| 7.我因别人对我的意见而感到不安  I feel insecure about others’ opinions of me | 1 | 2 | 3 | 4 | 5 |
| 8.别人因我有体重问题而歧视我  People discriminate against me because I’ve had weight problems | 1 | 2 | 3 | 4 | 5 |
| 9.没有体重问题的人很难理解我或和我交谈  It’s difficult for people who haven’t had weight problems to relate to me | 1 | 2 | 3 | 4 | 5 |
| 10.别人认为我的体重问题是因为我缺乏自我控制能力  Others will think I lack self-control because of my weight problems | 1 | 2 | 3 | 4 | 5 |
| 11.别人认为我要为我的体重问题自我责备  People think that I am to blame for my weight problems | 1 | 2 | 3 | 4 | 5 |
| 12.别人在我身旁时因我的体重问题而感到羞愧  Others are ashamed to be around me because of my weight | 1 | 2 | 3 | 4 | 5 |
